# Experimental Gingivitis Induces Systemic Inflammatory Markers in Young Healthy Individuals: A Single-Subject Interventional Study

**DOI:** 10.1371/journal.pone.0055265

**Published:** 2013-02-07

**Authors:** Jörg Eberhard, Karsten Grote, Maren Luchtefeld, Wieland Heuer, Harald Schuett, Dimitar Divchev, Ralph Scherer, Ruth Schmitz-Streit, Daniela Langfeldt, Nico Stumpp, Ingmar Staufenbiel, Bernhard Schieffer, Meike Stiesch

**Affiliations:** 1 Department of Prosthetic Dentistry and Biomaterials Science, Hannover Medical School, Hannover, Germany; 2 Department of Cardiology and Angiology, Hannover Medical School, Hannover, Germany; 3 Department of Medical Statistics, Hannover Medical School, Hannover, Germany; 4 Department of Operative Dentistry and Periodontology, Hannover Medical School, Hannover, Germany; 5 Institute for Microbiology, Christian-Albrechts-University Kiel, Kiel, Germany; Maastricht University Medical Center, The Netherlands

## Abstract

**Objectives:**

We here investigated whether experimental gingivitis enhances systemic markers of inflammation which are also known as surrogate markers of atherosclerotic plaque development.

**Background:**

Gingivitis is a low-level oral infection induced by bacterial deposits with a high prevalence within Western populations. A potential link between the more severe oral disease periodontitis and cardiovascular disease has already been shown.

**Methods:**

37 non-smoking young volunteers with no inflammatory disease or any cardiovascular risk factors participated in this single-subject interventional study with an intra-individual control. Intentionally experimental oral inflammation was induced by the interruption of oral hygiene for 21 days, followed by a 21-days resolving phase after reinitiation of oral hygiene. Primary outcome measures at baseline, day 21 and 42 were concentrations of hsCRP, IL-6, and MCP-1, as well as adhesion capacity and oxLDL uptake of isolated blood monocytes.

**Results:**

The partial cessation of oral hygiene procedures was followed by the significant increase of gingival bleeding (34.0%, *P*<0.0001). This local inflammation was associated with a systemic increase in hsCRP (0.24 mg/L, *P* = 0.038), IL-6 (12.52 ng/L, *P* = 0.0002) and MCP-1 (9.10 ng/l, *P* = 0.124) in peripheral blood samples between baseline and day 21, which decreased at day 42. Monocytes showed an enhanced adherence to endothelial cells and increased foam cell formation after oxLDL uptake (*P*<0.050) at day 21 of gingivitis.

**Conclusions:**

Bacterial-induced gingival low-level inflammation induced a systemic increase in inflammatory markers. Dental hygiene almost completely reversed this experimental inflammatory process, suggesting that appropriate dental prophylaxis may also limit systemic markers of inflammation in subjects with natural gingivitis. International Clinical Trials Register Platform of the World Health Organization, registry number: DRKS00003366, URL: http://apps.who.int/trialsearch/Default.aspx

## Introduction

Gingivitis and periodontitis are two distinct chronic inflammatory processes belonging to the spectrum of periodontal diseases of the oral cavity affecting the tooth supporting tissues in response to bacterial accumulation. In contrast to periodontitis, gingivitis is initiated only after a few days of inadequate oral hygiene procedures by local plaque deposits adjacent to the highly vascularised gingival tissues. Gingivitis is a superficial inflammatory affection and is not destructive towards the surrounding connective and bone tissues and completely declines with the initiation of adequate oral hygiene procedures. In gingivitis bleeding of the gums may occur even after gentle mechanical stimulation in severe cases or following tooth brushing and chewing [1]. In contrast, in susceptible subjects periodontitis is characterized by the irreversible loss of bone and periodontal ligament that is if untreated followed by tooth loss [2]. In addition to various epidemiological studies demonstrating a potential link between periodontitis and cardiovascular diseases [3] it has been demonstrated that the treatment of patients suffering from periodontitis reduces acute parameters of atherosclerosis and improves endothelial function [4], [5]. Equivalent data are not available for gingivitis, although the prevalence of moderate to severe periodontitis in western populations is 3% to 46% whereas gingivitis affects almost all individuals [6], [7], [8].

Inflammatory processes play a pivotal role in the pathogenesis of atherosclerosis and mediate the development of the disease, from initial leukocyte recruitment to sudden plaque rupture and subsequent myocardial infarction with often fatal outcome [9], [10]. Several systemic inflammatory markers have been identified as independent risk factors for cardiovascular diseases. The ability to measure levels of these inflammatory markers in the systemic circulation has provoked interest in their development as biomarkers or surrogate markers for potential use in risk assessment [11], [12]. In this regard, the acute phase reactant C-reactive protein (CRP) is a potential predictor of future atherosclerotic vascular disease, whereas cytokines and chemokines such as interleukin (IL)-6 or monocyte chemoattractant protein-1 (MCP-1, also known as CCL2) are established mediators of the chronic inflammatory process within the vascular wall in various experimental models (6–9). Activation of blood monocytes represents an early and crucial event in the development of atherosclerosis. Activated monocytes are prone to adhere more likely at inflamed sites of the endothelium to invade the arterial wall. Subsequently, they differentiate into foam cells in order to initiate atherosclerotic plaque growth. In this regard, chronic systemic inflammation has been shown to be linked to adverse cardiovascular outcomes [10]. Systemic or local infection may provide an additional inflammatory stimulus that could accelerate atherogenesis. However, the pathophysiological pathways between chronic extravascular infections and atherosclerosis remain unclear [3].

Although gingivitis is a highly prevalent chronic bacterial disease in susceptible children, adults and the elderly, persisting for decades in subjects, and is an essential precursor of periodontitis limited data are available today addressing detrimental systemic effects of gingivitis. Systemic effects of experimental or natural gingivitis have been rarely investigated and the existing studies did not show correlations between gingivitis and systemic levels of pro-inflammatory mediators or surrogate markers of atherosclerosis [13]. Recently, studies using broad-range frequency techniques using 454 pyrosequencing of 16S rRNA genes identified bacteria associated with gingivitis in atherosclerotic plaques and potentially highlight a mechanism by which low-level gingival inflammation may also affect cardiovascular diseases [14].

We now report the results of an intervention study using a single-subject design with an intra-individual control. Experimentally induced gingivitis in young, healthy volunteers was initiated by termination of oral hygiene and resolved after restart of oral hygiene to determine the effects and causal association of this highly prevalent chronic inflammatory oral disease on circulating inflammatory markers which in some studies have been suggested as surrogate markers of atherosclerotic plaque development.

## Methods

### Study Design

**Figure 1:**
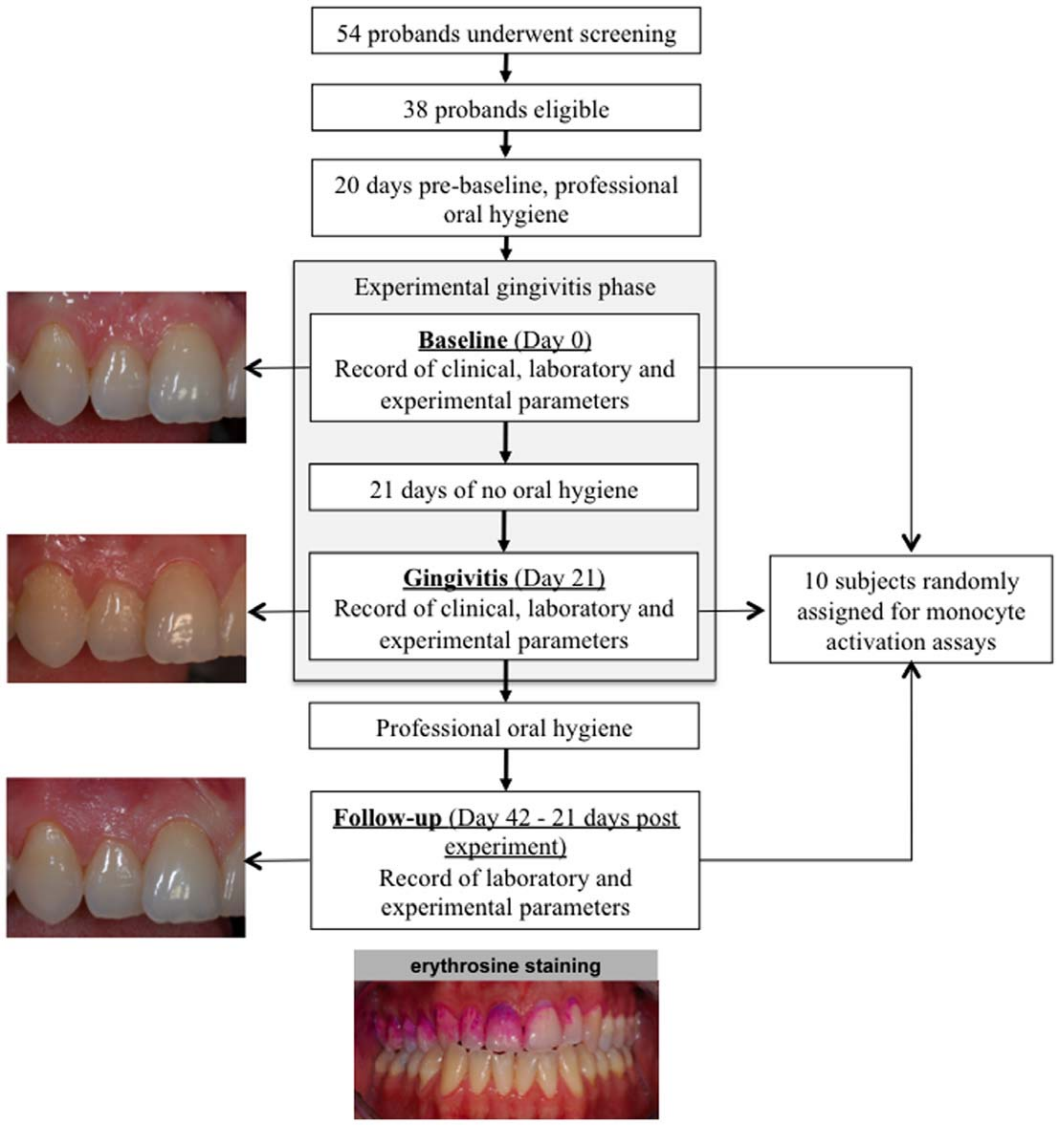
Flowchart of the study design from screening to follow-up. After a baseline visit, eligible volunteers were scheduled for intensive professional tooth cleaning and oral hygiene instructions to remove all biofilm deposits before starting the experimental gingivitis phase. After 21-days of cessation from any oral hygiene procedures in the upper right maxilla massive bacterial biofilms has been formed on the selected tooth hard substances, which are visible to the naked eye as yellowish plaques. Plaque covering of the tooth was routinely documented after erythrosine staining. The professional removal of all bacterial deposits and the restart of oral homecare terminated the experimental gingivitis phase. At baseline, day 21 (gingivitis) and 21 days after completion of the experimental gingivitis phase (follow-up) blood samples were obtained for serum parameters and monocyte activation assays.

Over a period of 42 days, it was our goal to evaluate the effects of oral hygiene cessation and restart on surrogate markers of atherosclerotic plaque development and activation of blood monocytes in young healthy individuals (Fig. 1). Oral hygiene cessation was selected as the intervention for the present study because of the high reproducibility of the following inflammatory responses in the gingival tissues. This model was introduced by Löe et al. as the “Experimental Gingivitis Model” in 1965 [15]. The primary outcome was the in-group differences between baseline and day 21 in clinical, laboratory and experimental parameters of inflammation during the study. Participants were selected according to the following inclusion criteria: (1) 20–30 years of age, (2) non-smokers, (3) no clinical signs of gingival inflammation (redness, swelling, bleeding) at ≥90% sites observed, (4) no probing pocket depth >3 mm at any site and (5) no alveolar bone loss. Exclusion criteria were: (1) presence of systemic diseases (e.g., diabetes mellitus or cardiovascular, kidney, liver or lung disease) (2) pregnancy or breastfeeding, (3) history of drug abuse, (4) allergic diathesis, (5) medications, in particular currently ingestion of non-steroidal or steroidal anti-inflammatory drugs, analgesic or antibiotics within 3 months before entering the study, (6) untreated carious lesions and/or insufficient restorations, implants, crowns at teeth in the maxillary right quadrant and (7) mouth breathing. All participants gave written informed consent. The study was performed in accordance with the Helsinki Declaration, approved by the ethical Committee of Hannover Medical School and registered at the International Clinical Trials Register Platform of the World Health Organization (http://apps.who.int/trialsearch/Default.aspx, ID: DRKS00003366).

Volunteers, which were addressed by public announcement in the Hannover area, who met the inclusion criteria were scheduled for a dental and periodontal screening appointment and full medical and dental histories were collected. All participants had a college degree. After inclusion in the study, all participants received a professional scaling and polishing of all tooth surfaces to remove supra- and subgingival plaque, stain and calculus 14 days prior to the start of the experimental gingivitis phase. The intervention was initiated at baseline when all participants refrained from any oral hygiene at seven upper teeth of the right maxilla. This model of limited inflammation has been described previously and was used as a model for an inflammatory status in the oral cavity that may represent a inflammatory condition affecting the majority of cases in the general population [16]. The subjects were clinically examined and 20 ml peripheral venous blood was sampled at baseline as well as on day 21 (experimental gingivitis) and at the follow-up appointment 21 days after termination and 42 days after initiation of the experimental gingivitis phase. Professional tooth cleaning and topical fluoride application was applied at day 21 of the experimental gingivitis phase (Fig. 1). Hereby at day 21 the intervention was terminated and during the following 21 days rigorous oral hygiene procedures were applied in order that the subjects served as their own control.

### Periodontal Clinical Examination

Periodontal data were recorded by three experienced and calibrated dentists at baseline and day 21. The data included the presence or absence of supragingival dental plaque using a modification of the Silness-Löe plaque index (PI) at the buccal and oral sites of the selected teeth and the observable inflammatory changes of the gingival tissues by the gingival index (GI) at four sites per tooth [17], [18]. The volume of the gingival crevicular fluid (GCF) was determined after gentle drying of the tooth for ten seconds followed by insertion of a filter paper strip (Periopaper, Pro Flow Incorporated) for 30 seconds into the gingival sulcus at four sites of the upper right first premolar. The GCF was measured with a calibrated Periotron 6000 gingival fluid meter (Pro Flow Incorporated) and expressed in Periotron units (PU). Gingival bleeding on probing was also recorded (BOP) with a pressure-calibrated probe (TPS probe, Vivacare) at four sites of all selected tooth sites: mesio-buccal, disto-buccal, mesio-oral and disto-oral. All measurements were recorded at four identical sites per tooth. The averaged scores for supragingival plaque and gingival inflammation (the sum of indexed scores divided by the total number of recorded sites), the amount of gingival crevicular fluid (the sum of the Periotron units per tooth divided by the number of sites) and the score for gingival bleeding on probing (the number of sites with gingival bleeding divided by the total number of sites, multiplied by 100) were calculated for each subject.

### Species-specific Detection of Bacteria in Plaque Samples

Supragingival plaque was collected with sterile paper strips (VDW) at day 21 from all participants at the upper teeth of the right maxilla. Total genomic DNA was isolated using the QIAamp DNA Mini kit (Qiagen) according to the manufacturer’s protocol for Gram-positive bacteria, but including a mechanical disruption step with a Precellys 24 bead mill (Bertin Technologies) prior to the first step of the protocol. Total genomic DNA was used as template for PCR-based detection of ten representative bacterial species commonly found in supragingival plaque samples belonging to different complexes according to Socransky et al. and Haffajee et al. [19], [20]. The ‘red complex’, comprising of *Porphyromonas gingivalis*, *Tannerella forsythia*, and *Treponema denticola*, has been identified as the ‘disease-related’ complex. For supragingival plaque it has been shown that members of the red and orange complexes (e.g. *Fusobacterium nucleatum*) were most frequently associated with inflamed sites [19]. In the remaining clusters (purple, green and yellow) were moderately pathogenic species, including initial and early colonizers. The species-specific primers for the selected microorganisms are listed in table 1. All PCR reactions were performed on a TProfessional thermocycler (Biometra) in a total reaction volume of 20 µl. The reaction mix contained 2 µl of genomic DNA as template, 200 nM of each specific primer, 1x PCR buffer (including 1.5 mM magnesium chloride; Qiagen), 1.5 U HotStar Taq polymerase (Qiagen), 200 mM of each dNTP (Roth) and PCR-grade water (Roche). The thermal cycling protocol for each primer set was as follows: Initial denaturation at 95°C for 15 min; 40 amplification cycles consisting of denaturation at 94°C for 30 s, annealing at 55°–68°C for 45 s, elongation at 72°C for 1 min; final extension at 72°C for 10 min. 5 µl of each amplification reaction were analyzed by 1% agarose gel electrophoresis. A clear single band of expected size (table 1) was evaluated as positive detection signal and respective PCR products were subsequently sequenced.

**Table 1:** Species-specific primers used to identify ten different bacterial species in plaque samples of experimental gingivitis at day 21 of the study.

Bacterial species	Primer	Annealing temperature	Expected amplicon size (bp)	Reference
*Prevotella intermedia*	F: 5′-CGTGGACCAAAGATTCATCGGTGGA-3′	64°C	259	[40]
	R: 5′-CCGCTTTACTCCCCAACAAA-3′			
*Fusobacterium nucleatum*	F: 5′-AGAGTTTGATCCTGGCTCAG-3′	60°C	360	[41]
	R: 5′-GTCATCGTGCACACAGAATTGCTG-3′			
*Streptococcus oralis*	F: 5′-TCCCGGTCAGCAAACTCCAGCC-3′	66°C	374	[42]
	R: 5′-GCAACCTTTGGATTTGCAAC-3′			
*Streptococcus sanguinis*	F: 5′-GGATAGTGGCTCAGGGCAGCCAGTT-3′	70°C	313	[42]
	R: 5′-GAACAGTTGCTGGACTTGCTTGTC-3′			
*Capnocytophaga* *sputigena*	F: 5′-AGAGTTTGATCCTGGCTCAG-3′	55°C	185	[43]
	R: 5′-GATGCCGCTCCTATATACCATTAGG-3′			
*Eikenella corrodens*	F: 5′-CTAATACCGCATACGTCCTAAG-3′	60°C	688	[44]
	R: 5′-CTACTAAGCAATCAAGTTGCCC-3′			
*Tannerella forsythia*	F: 5′-GCGTATGTAACCTGCCCGCA-3′	60°C	641	[44]
	R: 5′-TGCTTCAGTGTCAGTTATACCT-3′			
*Porphyromonas gingivalis*	F: 5′-AGGCAGCTTGCCATACTGCG-3′	60°C	404	[44]
	R: 5′-ACTGTTAGCAACTACCGATGT-3′			
*Treponema denticola*	F: 5′-TAATACCGAATGTGCTCATTTACAT-3′	60°C	316	[44]
	R: 5′-TCAAAGAAGCATTCCCTCTTCTTCTTA-3′			
*Veillonella parvula*	F: 5′-GAAGCATTGGAAGCGAAAGTTTCG-3′	57°C	623	[45]
	R: 5′-GTGTAACAAGGGAGTACGGACC-3′			
*Aggregatibacter actinomycetemcomitans*	F: 5′-TAGCCCTGGTGCCCGAAGC-3′	68°C	557	[46]
	R: 5′-CATCGCTGGTTGGTTACCCTCTG-3′			

### Cardiovascular and Laboratory Assessment

Carotid intima-media thickness (IMT) was assessed at baseline and at day 42 by B-mode duplex sonography using a 10.5-MHz linear transducer. IMT was measured at the common carotid artery approximately 10 mm proximal to the bifurcation. The mean values from both sides are reported. Non-invasive blood pressure measurement was performed at baseline and at day 42 in seated position by Riva-Rocci/Korotkow’s method. A standard 2D-Doppler transthoracic echocardiogram was performed at baseline and at day 21. Left ventricular function was assessed qualitatively. Valvular status was quantified by color and continuous-wave Doppler flow.

Serum and plasma samples were obtained at baseline, day 21 of the experimental gingivitis phase and at the follow-up re-evaluation at day 42. All samples were collected between 7.45 and 8.30 a.m. after a period of at least eight hours of fasting. Blood counts and measurements of lipid levels were done by standard laboratory testing. Serum concentrations of hsCRP were determined by immunonephelometry (detection limit 0.23 mg/L, Cardiophase hsCRP, Siemens). Serum levels of IL-6 (detection limit 0.70 pg/mL) and MCP-1 (detection limit 5.0 pg/mL) were measured by enzyme-linked immunosorbent assay (Quantikine HS, R&D Systems) with the help of a plate reader (µQuant; Bio-Tek Instruments).

### Cell Culture

Human endothelial cells (human umbilical vein endothelial cells = HUVECs) were obtained from Lonza. Cells were cultured in endothelial cell growth medium (EGM-2; Lonza) supplemented with 2% FCS and growth factors and cultured on gelatin coated cell culture plates. Cells between passage 2 and 4 were used for experiments. Human leukocytes were isolated from the blood of the subjects. Erythrocytes were removed by hemolysis (155 mM NH_4_Cl, 10 mM KHCO_3_, 0.01% ethylenediaminetetraacetic acid; for 10 min) and cells were resuspended in X-vivo 15 medium (Lonza). Monocytic cells were enriched by plastic adherence over night, non-adherent cells were removed.

### Cell Adhesion

50,000 monocytes/well were seeded on a confluent monolayer of HUVECs in a 96-well plate in EGM-2 medium (Lonza). Adhesion was carried out for two hours at 37°C and 5% CO_2_. Non-adherent cells were removed by 2 washing steps with PBS. Adherent cells were visualized per well in triplicates using a cell culture microscope (CKX31; Olympus) and captured with a digital camera (C-5060; Olympus). Adherent monocytes on a monolayer of endothelial cells were quantified using computer-assisted image analysis software (ImageJ 1.43 h, NIH).

### Foam Cell Formation

50,000 monocytes/well were seeded in a 96-well plate in X-vivo 15 medium (Lonza) and monocyte-derived macrophages were obtained by plastic adherence for 4 days. Macrophages were stimulated for 4 hours with oxLDL (10 µg/ml). After treatment, cells were washed twice with PBS, fixed with formalin and dehydrated with 2-propandiol. Cellular lipids were stained with oil red O (Sigma) for 30 minutes. Oil red O-stained cells were visualized in three randomly chosen microscopic fields per well using a cell culture microscope (CKX31; Olympus) and captured with a digital camera (C-5060; Olympus). Percentage of Oil red O-positive cells was quantified using computer-assisted image analysis software (ImageJ 1.43 h, NIH).

### Statistical Analysis

The statistical analysis was conducted with the statistical programming language R for the hypothesis testing and for the construction of graphs. The local two-sided Type-I-errors were set to 5% resulting in an explorative data analysis. In all comparisons of baseline values against gingivitis status and gingivitis status against follow-up, two-sided Wilcoxon signed-rank tests for paired samples were used. The exact *P*-values and exact confidence intervals were calculated for the monocytes adhesion assays and foam cell formations. Due to the exploratory character of the study all *P*-values were not adjusted for multiplicity. Results are presented as median differences with two-sided 95% confidence intervals as well as corresponding two-sided *P*-values. Post-hoc power calculations were based on the two-sample t test for paired data using a two-sided significance level of 0.05. The estimators for the mean differences and standard deviations were calculated from the present data.

## Results

### Subjects Characteristics

52 individuals were screened at the Hannover Medical School of Dentistry; of those 38 met the inclusion criteria and 37 subjects completed the clinical trial (Fig. 1). First subject in was 1^st^ March 2010, last subject out was 28 July 2010. One volunteer showed clinical signs of congenital heart valve insufficiency and was withdrawn from the study. 37 non-smoking young and healthy volunteers (mean age 23.35±3.64 years) were enrolled in this study free of any traditional risk factor or inflammatory disease. The baseline characteristics of the subjects are presented in table 2.

**Table 2:** Baseline characteristics of the patients.

Characteristic		Study Group	Reference intervals*
		(N = 37)	(Male/Female)
Age - yr		23.4±3.6	
Male/Female sex - no. (%)		6/31 (16.2/83.8)	
Smoking status - no. (%)			
	Never smoked	31 (83.8)	
	Former Smoker¶	6 (16.2)	
	Current smoker	0 (0)	
Family history of cardiovascular disease - no. (%)		3 (8.1)	
Race or ethnic group - no. (%)§			
	White	31 (83.8)	
	Asian	6 (16.2)	
	Black	0 (0)	
Body-mass index§§		22.9±4.5	18.5–24.9
Blood pressure - mm Hg			
	Systolic	124.0±12.0	<140
	Diastolic	81.0±8.8	<90
Cholesterol fractions Male/Female (SD) - mmol/l			
	S-cholesterol	5.9±1.8/6.2±2.1	3.3–6.2/3.3–6.2
	S-HDL-cholesterol	1.8±0.3/2.4±0.8	>1.0/>1.2
	S-LDL-cholesterol	4.0±1.4/4.0±1.4	1.8–4.5/1.7–4.2
Haemogram			
	haemoglobin - g/dl	13.2±1.4	12.0–17.5
	erythrocyte count - ×10^12^/liter	4.6±0.5	4.0–5.9
	thrombocyte count - ×10^9^/liter	261.5±55.9	150.0–450.0
	neutrophile count - ×10^9^/liter	5.8±0.8	1.1–8.8
	eosinophile count - ×10^9^/liter	0.2±0.1	<0.5
	basophile count - ×10^9^/liter	0.5±0.3	0.0–1.0
	haematocrit - %	41.3±3.2	36.0–50.4

¶ Smoking was terminated at least 12 months prior to the start of the study.

§ Race was self-reported.

§§ Body-mass index is defined as the weight in kilograms divided by the square of the hight in meters.

* All parameters fall within the reference intervals or are designated as „normal“.

As safety parameter and as marker of pre-existing atherosclerosis, the intima-media thickness of the left and right common carotid artery was determined. At baseline and after 42 days no differences were found (baseline: 0.55±0.16 mm, 21 days: 0.54±0.18 mm, *P* = 0.160). Neither blood pressure nor serum cholesterol fraction showed significant differences when baseline was compared to day 21. Moreover, echocardiographical assessment of left ventricular function and valvular status was performed at baseline and at day 21 and showed no differences within the individuals.

### Markers of Experimental Gingivitis

**Figure 2:**
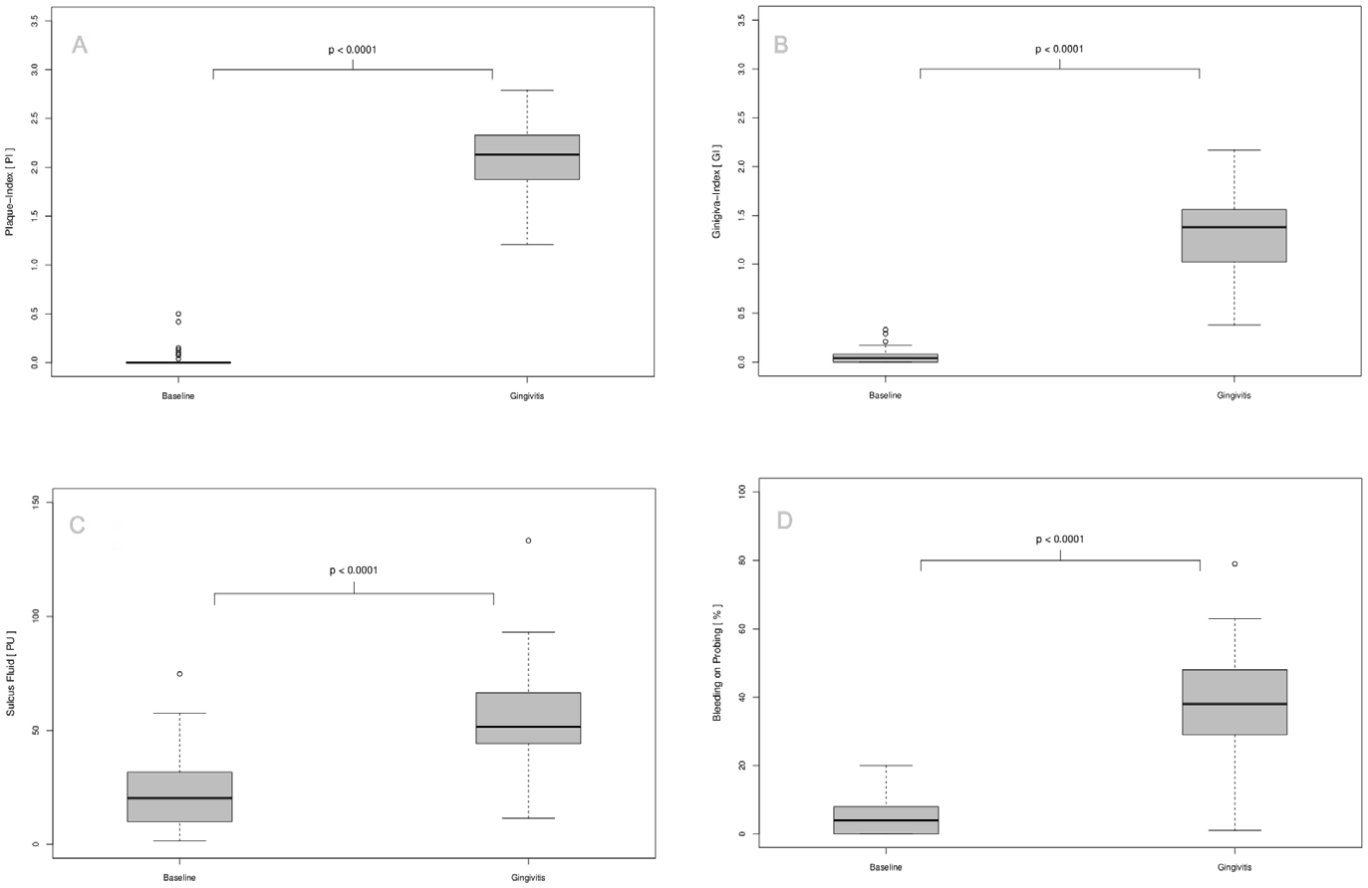
Characterization of oral clinical parameters at baseline and after 21 days of experimental gingivitis. The box-plots illustrate the plaque accumulation on tooth hard-substances during the 21-days non-brushing period followed by the progression of three selected clinical inflammatory parameters documenting the inflammatory status of the gingival tissues. The time interval and clinical observations are characteristic for an inflammatory lesion denoted as gingivitis.

All volunteers developed experimental gingivitis as determined by all recorded parameters. The cessation of oral hygiene for 21 days in the upper right maxilla was followed by the accumulation of bacterial deposits on the tooth surfaces adjacent to the gingival tissues surrounding the teeth (absolute difference 2.04 PI, 95% CI, 1.91 to 2.17; *P*<0.0001) and is depicted in Figure 2A. This plaque formation was followed by the induction of inflammatory changes of the gingival tissues that became obvious by redness and swelling of the marginal gingiva categorized by the Gingival Index (absolute difference -1.29 GI, 95% CI, 1.11 to 1.40; *P*<0.0001, Fig. 2B). The accumulating inflammatory processes in the marginal gingival tissues between baseline and day 21 were associated with an increased volume of the gingival sulcus fluid (absolute difference 33.20 PU, 95% CI, 25.13 to 40.12; *P*<0.0001, Fig. 2C) and increased frequencies of bleeding on probing (absolute difference 34.00%, 95% CI, 28.00 to 39.00%; *P*<0.0001, Fig 2D).

### Verification of Bacterial Plaque Composition

**Figure 3:**
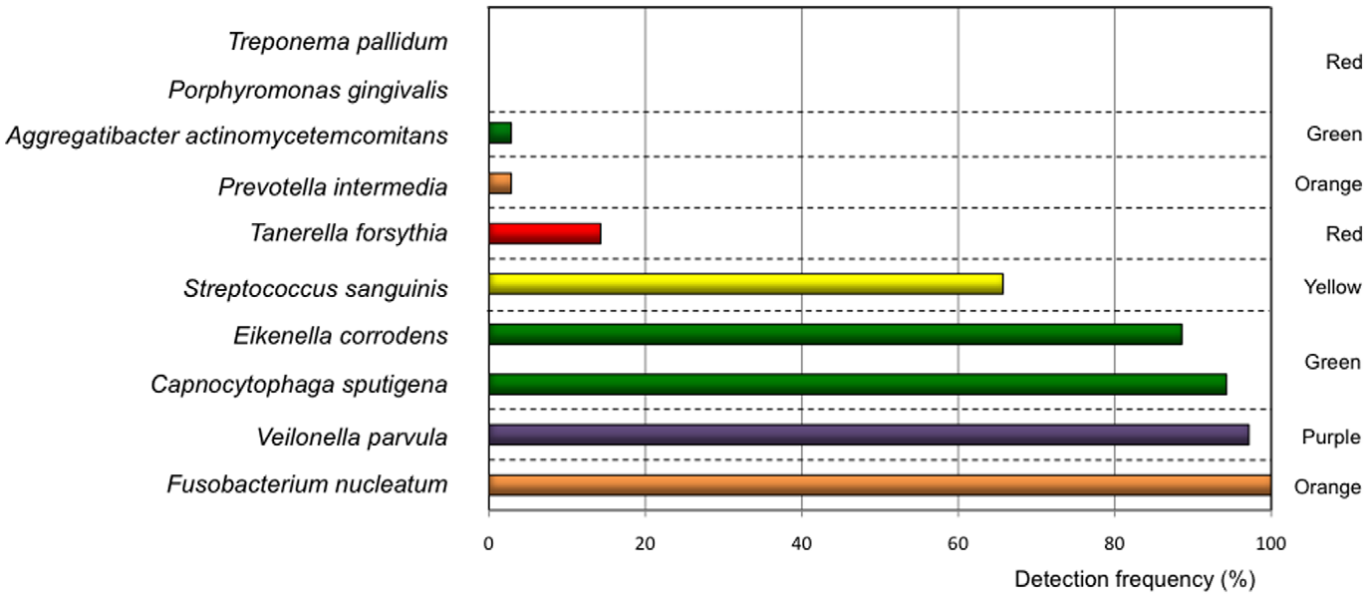
Species-specific detection of bacteria in 21-day old dental biofilms. The box-plots represent the PCR-based detection frequency of ten representative bacterial species within the study population of 37 subjects. The colors of the bars comply with the color codes of the bacterial complexes introduced by Socransky et al. [20].

Aiming to identify ten bacterial species belonging to different clusters introduced by Haffajee et al. [19] and commonly found in sub- or supragingival plaque a species-specific PCR amplification was performed. We identified bacteria belonging to the red-complex in low detection frequencies within the study population of 37 subjects, *Porphyromonas gingivalis* (0.0%), *Tanerella forsythus* (13.5%) and *Treponema pallidum* (0.0%). Whereas *Fusobacterium nucleatum* (orange complex) was detected in every plaque sample from all test persons (100.0%). *Veilonella parvula* (purple complex) was observed in 97.3%, *C. sputigena* (green complex) in 94.1%, *E. corrodens* (green complex) in 88.6% and *S. sanguinis* (yellow complex) in 65.7% of all samples (Fig. 3). In summary, we could detect seven from ten representative bacterial species commonly found in plaque samples in high frequency across the whole group of participants. Whereas species from the red-complex associated with periodontitis, were absent or in low frequency.

### Surrogate Markers of Atherosclerosis

**Figure 4:**
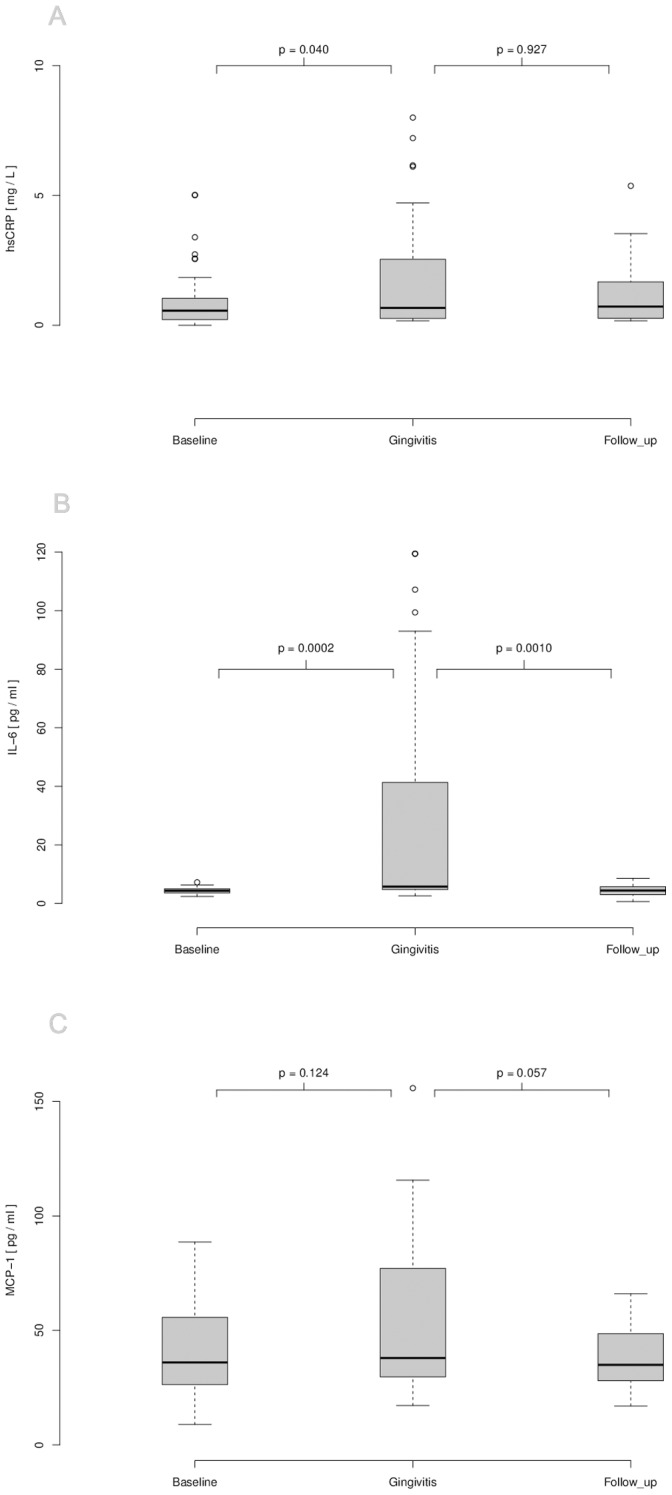
Characterization of serum parameters of inflammation at baseline, experimental gingivitis and follow-up. (A) Serum of participants was analyzed for hsCRP by immunonephelometrie. Plasma of participants was analyzed for IL-6 (B) and MCP-1 (C) by ELISA. Serums of participants were analyzed for leucocyte (D) and monocyte (E) cell counts by flow cytometry. *P*-values were calculated using two-sided Wilcoxon signed-rank tests for paired samples.

Analysis of plasma samples revealed enhanced levels of circulating markers of inflammation when experimental gingivitis was induced. First of all, hsCRP mean difference was found to be significantly elevated under experimental gingivitis conditions 0.24 mg/L, 95% CI 0.01 to 1.20; *P* = 0.038 (Fig. 4A). At follow-up, hsCRP levels tended to decline but were still slightly enhanced (median difference −0.02 mg/lL, 95% CI 0.28 to 0.71; *P* = 0.927. Likewise, we observed enhanced circulating levels of the inflammatory mediators for IL-6 (Median difference 12.52 ng/l, 95% CI 1.25 to 43.85; *P* = 0.0002) and MCP-1 (Median difference 9.10 ng/l, 95% CI −3.70 to 28.65; *P* = 0.124) (Fig. 4B and C) between baseline and gingivitis, which both returned to baseline levels at the follow-up appointment (Median difference for IL-6 −12.51 ng/l, 95% CI −44.65 to −1.36; *P* = 0.001 and median difference for MCP-1 −12.10 ng/l, 95% CI −27.00 to 0.50; *P* = 0.057). Compared to baseline blood leukocyte and monocyte cell counts were increased upon experimental gingivitis (median differences 0.45×10^9^/L, 95% CI −0.049 to 0.949, *P* = 0.0001, respectively 0.010×10^9^/L, 95% CI 0.099 to 0.150, *P* = 0.068) and did not yet decline back to baseline levels at follow-up (mean differences for leukocytes 0.15×10^9^/L, 95% CI −0.499 to 0.700, *P* = 0.404, and for monocytes 0.000×10^9^/L, 95% CI −0.049 to 0.100, *P* = 0.591 (Fig. 4D, E).

### Monycyte Activation Assays

**Figure 5:**
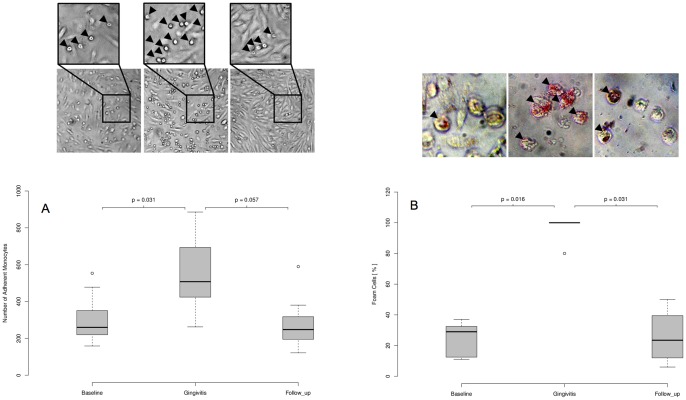
Monocyte adhesion assays and foam cell formation at baseline, gingivitis and follow-up. Blood monocytes were isolated from the blood of the volunteers at baseline (day 0), gingivitis (day 21) and follow-up (day 42) and subjected to ex vivo activation assays. (A) Number of adherent monocytes on cultured endothelial cells (HUVECs). Arrowheads in the enlarged picture detail indicate adherent monocytes. (B) Percentage foam cell formation after stimulation with oxLDL (10 µg/ml). Arrowheads indicate oil red O-positive foam cells. Representative pictures are shown. *P*-values were calculated using two-sided Wilcoxon signed-rank tests for paired samples.

Monocytes were isolated from the blood of ten randomly assigned study participants and subsequently subjected to ex vivo cell culture assays verifying monocyte activation. Of note, experimentally induced gingivitis led to significantly enhanced adherence of monocytes on endothelial cells (Fig. 5A) as well as to augmented oxLDL-uptake and foam cell formation (Fig. 5B). Both processes returned to baseline levels at day 42. Of note, monocyte activation is known to be crucially involved in the development of atherosclerosis and thereby fundamental to the etiology of coronary artery disease.

A post-hoc power analysis using observed mean differences and SDs demonstrated that this study had more than 80% power to detect differences between baseline and gingivitis for all parameters, except for MCP-1 (11% power), hsCRP (62% power) and Leucocyte counts (55% power).

## Discussion

The present interventional study showed that even in healthy young individuals, experimentally induced gingivitis as low-level extravascular oral bacterial infection lead to an acute systemic inflammation with enhanced systemic levels of CRP, IL-6, MCP-1 and activated monocytes known as markers and mediators of vascular atherosclerotic disease. This inflammatory process seems to be almost completely reversible by appropriate dental hygiene.

Gingivitis is a chronic local infection affecting the tooth surrounding tissues, caused by bacteria of the dental plaque (e.g. *Streptococcus oralis, Veilonella ssp.* etc.) and is accompanied by a local inflammatory response of the host. The oral inflammatory response is, in contrast to periodontitis, limited to the soft gingival tissues not affecting the bone. Moreover, gingivitis differs from periodontitis with respect to its microbial composition and inflammatory responses [21]. In consequence, it was not expected at the beginning of the study to observe systemic effects from gingivitis, although they have already been shown for periodontitis [5]. Recently, Wahaidi et al. [13] was not able to show an association of experimental gingivitis and systemic CRP and IL-6 levels in a population of healthy adults aged 18 to 30 years. These experiments were in accordance with a study of natural gingivitis by Wohlfeil et al. [22]. This group also showed no correlation between serum CRP levels and the degree of gingivitis. In contrast, elevated levels of serum IL-6 were found in patients suffering from type 2 diabetes and natural gingivitis compared to patients with diabetes and healthy gingival conditions [23]. The different observations between these studies and the present study may be explained at least in part by the young healthy participants of the present study, which do not have any traditional risk factors for cardiovascular diseases. In addition, a very sensitive method for the detection of CRP in serum was used.

Most likely, the observed gingival inflammatory processes, which was induced in the present study affect approximately 75% of Western populations [1]. In contrast to periodontitis, gingivitis affects children, adults and elderly in nearly equal ratios [8]. Clinically, this inflammatory process becomes obvious by bleeding during brushing the teeth, normally on a limited number of teeth. For the present study this clinical appearance was tried to mimick by inducing the inflammatory processes also just on a limited number of teeth, here seven, during a 21-days plaque accumulation phase. The inflammatory reactions of the gingival tissues are only mild and clinically nearly inconspicuous to the untrained eye. We studied well-characterized young healthy volunteers that yet did not show any traditional risk factor for cardiovascular diseases. Thus, we are assured that the observed systemic changes are a consequence of the local gingival inflammatory processes. However, the participants did not respond equally and showed inter-individual differences regarding the amount of dental plaque load, clinical signs of inflammation and serum markers of inflammation.

To further address the oral inflammatory status, we analyzed the presence of ten different species belonging to variable complexes according to Haffajee et al. [19], in which potential pathogens spread across different clusters. Bacterial species predominantly associated with subgingival lesions of destructive periodontitis were if at all in very low numbers present in the developing supragingival plaques of the present study [24], [25]. Only *Fusobacterium nucleatum,* a so called ‘bridge-bacterium’ between health and disease, could be detected in high frequency in all plaque samples. Generally periopathogens, namely *Porphyromonas gingivalis*, are in the focus of research aimed to identify pathological mechanisms between periodontitis and systemic diseases [26]. The present study demonstrated that systemic effects could also be induced by low-level gingival inflammation and by bacterial plaques that do not harbour traditional periopathogens like *Porphyromonas gingivalis*. Hence, the present study is the first report that demonstrates adverse effects of local experimentally induced gingival inflammation on circulating serum levels of CRP, IL-6 and MCP-1 and activation of monocytes. These results demonstrated an enhanced systemic inflammatory status of the subjects during the course of the study. Future experiments are warranted to evaluate if natural gingivitis also enhances systemic inflammatory markers and the risk for atherosclerotic diseases. Elevated CRP, IL-6 and MCP-1 serum levels could serve as a direct marker of the inflammatory activity in the subjects’ gingiva. The serum concentrations of hsCRP and IL-6 during the state of gingivitis reached levels, which were previously shown to be associated with high cardiovascular event rate i.e. myocardial infarction [27], [28]. The broad concordance between clinical markers of gingival inflammation and systemic inflammatory markers and monocyte activation suggests a relationship between the local and systemic recations. Only one epidemiological study has been published aimed to identify associations between gingival inflammation and angina pectoris, however, the reported results are critical due to various confounding factors and unpredictable outcomes of self-reported data sampling [29]. For the present study the clinical parameters of experimental gingivitis were not followed after day 21 of the experimental gingivitis phase, because multiple studies have shown that the clinical signs of gingivitis decline within seven days after the re-initiation of oral hygiene procedures in a group of subjects highly familiar with oral hygiene [30], [31].

A potential limitation of this study is the sample size of 37 volunteers. However, the single-subject design with the subject as its own control is highly sensitive to detect individual differences. The here presented findings with young healthy individuals could not be generalized since elder subjects have an overall higher risk burden. Nevertheless, we especially have chosen young healthy subjects with no cardiovascular risk factors, to study systemic effects of experimental gingivitis. Thus, this approach offers the best prospect to uncover a potential relationship between local oral inflammation in gingivitis and systemic effects on surrogate markers of atherosclerosis. Definitely, larger randomised multicenter trials with elder subjects are necessary to investigate the hypothesis whether oral low-level gingivitis increases the risk for atherosclerosis or even enhances cardiovascular events.

The mechanisms by which local inflammatory processes of the oral cavity affect systemic conditions remain largely unknown and future experimental studies are warranted. Gingivitis involves bacterial infection with a range of Gram-positive and Gram-negative bacteria that invade the superficial gingival tissues [32]. During progression of the gingival disease, the epithelium becomes ulcerated to expose the underlying connective tissues and blood capillaries and facilitates entry of biofilm organisms or their products (e.g. bacterial heat-shock proteins) to the circulation during eating or tooth brushing [33]. Pathogens which have entered the bloodstream can directly invade blood vessel walls and atherosclerotic plaques [34], [35], [36]. Finally both, local oral infection and circulating infection products lead to an increase in the levels of circulating cytokines, chemokines and acute phase proteins, which are known to be associated with an increased cardiovascular event rate. The present study is the first report that demonstrated elevated serum levels of CRP, IL-6 and MCP-1 in the course of experimental gingivitis. Of interest, even gingival tissues were found to synthesize CRP [37]. Other inflammatory diseases, such as lupus erythematosus and rheumatoid arthritis are also associated with an increased cardiovascular risk supporting a mechanistic link between local extravascular inflammation and cardiovascular diseases [38], [39].

It will be a task for future studies to identify the mechanisms that may account for the observed individual levels of local and systemic host responses. The potential confirmation that natural gingival inflammatory processes are an extravascular stimuli for systemic inflammation and atherogenesis will have great public health implications.

## Supporting Information

Checklist S1 (DOC)

Protocol S1 (DOC)
