# Supplementary material for: Experimental Gingivitis Induces Systemic Inflammatory Markers in Young Healthy Individuals: A Single-Subject Interventional Study
Source: PLoS One. 2013 Feb 7;8(2):e55265. doi: 10.1371/journal.pone.0055265 (PMC3567060; doi:10.1371/journal.pone.0055265)
Supplement: Protocol S1 — (DOC) [file pone.0055265.s002.doc]

**Die Expression von antimikrobiellen Peptiden in der Gingiva während verschiedener Stadien einer experimentell induzierten, natürlichen Zahnfleischentzündung**

Kerstin Schulze^1^, Ingmar Staufenbiel^1^, Regine Gläser^2^, Jens-Michael Schröder^2^ & Jörg Eberhard^3^

^1^Klinik für Zahnerhaltung und Parodontologie, Medizinische Hochschule Hannover, Hannover,^2^Klinik für Dermatologie, Universitätsklinikum Schleswig-Holstein, Campus Kiel

# **Ansprechpartner:**

# Prof. Dr. Jörg Eberhard, MME

# Klinik für Zahnerhaltungskunde und Parodontologie

# Medizinische Hochschule Hannover

# Carl-Neuberg-Str. 1

# 30625 Hannover

# Tel. 0511 532 4336

# Fax. 0511 532 4818

# Email. Eberhard.joerg@mh-hannover.de

Prof. Dr. med. dent. Jörg Eberhard, MME

Oberarzt der Klinik

für Zahnerhaltungskunde und Parodontologie

# **Ziel und Notwendigkeit der Untersuchung**

Das Ziel der geplanten Studie besteht darin, die Expression der antimikrobiellen Peptide hBD-2, hBD-3, RNase-7 und Psoriasin im oralen Epithel während verschiedener Stadien einer natürlichen reversiblen Entzündungsreaktion zu untersuchen.[[1]](#footnote-2) Antimikrobielle Peptide wurden auf unterschiedlichen muko-epithelialen Oberflächen des menschlichen Körpers nachgewiesen und sind für die angeborene Immunantwort von großer Bedeutung (Chung et al. 2007). Für die geplante Untersuchung soll die Expression der antimikrobiellen Peptide am Modell der experimentellen Gingivitis untersucht werden. Die Untersuchung erlaubt erstmalig die Expression antimikrobieller Peptide, und damit einen Teilbereich der angeborenen Immunantwort, im Verlauf einer zunehmenden natürlichen Entzündungsreaktion zu beurteilen. Die Ergebnisse werden wichtige Daten zu den physiologischen Bedeutungen der antimikrobiellen Peptide liefern.

## **Darstellung der bisher zur Fragestellung durchgeführten Untersuchungen an Tieren und Menschen einschließlich der dabei verwendeten Techniken und dabei gewonnenen Erfahrungen**

Orale Epithelzellen können antimikrobielle Peptide synthetisieren und tragen damit direkt zur Abwehr oraler Pathogene bei (Chung et al. 2007; Diamond et al. 2001; Eberhard et al. 2008). Die Expression antimikrobieller Peptide in Biopsien gesunder und erkrankter oraler Gewebe wird kontrovers diskutiert. Während Studien zeigten, dass die Expression antimikrobieller Peptide vom Entzündungszustand der Gewebe abhängig ist (Bissell et al. 2004), konnten andere Studien diese Beobachtung nicht bestätigen (Dommisch et al. 2005; Dunsche et al. 2001). Die Interpretation der Untersuchungen wird durch eine nur ungenügend bestehende Möglichkeit, den Entzündungszustand von Patientenbiopsien zu charakterisieren, erschwert. Damit ist zum heutigen Zeitpunkt die physiologische Bedeutung antimikrobieller Peptide für die angeborene Immunantwort in der Mundhöhle nicht geklärt.

Das Modell der experimentellen Gingivitis erlaubt reversibel, eine entzündliche Reaktion der marginalen Gingiva zu induzieren. Durch die Messung klinischer Parameter lässt sich in diesem Modell die entzündliche Reaktion exakt quantifizieren (Eberhard et al. 2005).

## **Darstellung der Risiken für den Probanden**

Zu Beginn der Studie wird eine professionelle Zahnreinigung sowie eine ausführliche Mundhygieneinstruktion durchgeführt. Anschließend werden die Probanden angewiesen, die Mundhygiene an 6 Zähnen des Oberkiefers einzustellen. Alle weiteren Zähne können mechanisch gereinigt werden. Ab diesem Zeitpunkt akkumulieren entlang des Zahnfleischrandes orale Bakterien und führen individuell verschieden nach 4-7 Tagen zu einer Entzündung der benachbarten Gewebe (Ramberg et al. 2003). Mit der Wiederaufnahme der Mundhygiene am 14. Tag und der professionellen Zahnreinigung kommt es innerhalb weniger Tage zur vollständigen Abheilung der entzündlichen Veränderungen (Loe et al. 1965). Bleibende Schäden an den Zähnen oder dem Zahnfleisch bleiben nicht zurück.

In dem beschriebenen Zeitraum werden an den Tagen 0, 1, 3, 5, 7 und 14 und 21 Tagen Biopsien der marginalen Gingiva von den Probanden entnommen. Die Gewebestücke haben eine Größe von etwa 2x1,5 mm und werden an den in Abb. 1 gezeigten Stellen mithilfe eines Skalpells entnommen. Das Gewebe wird für diesen Zweck mit 0,2ml eines Lokalanästhetikums anästhesiert. Die allgemeinen Risiken für eine Lokalanästhesie bestehen und es ist der Proband darauf aufmerksam zu machen, dass nach einer Lokalanästhesie für 2-3 Stunden kein Fahrzeug geführt werden kann. Innerhalb von 7 Tagen ist die entstandene Wunde vollständig abgeheilt.


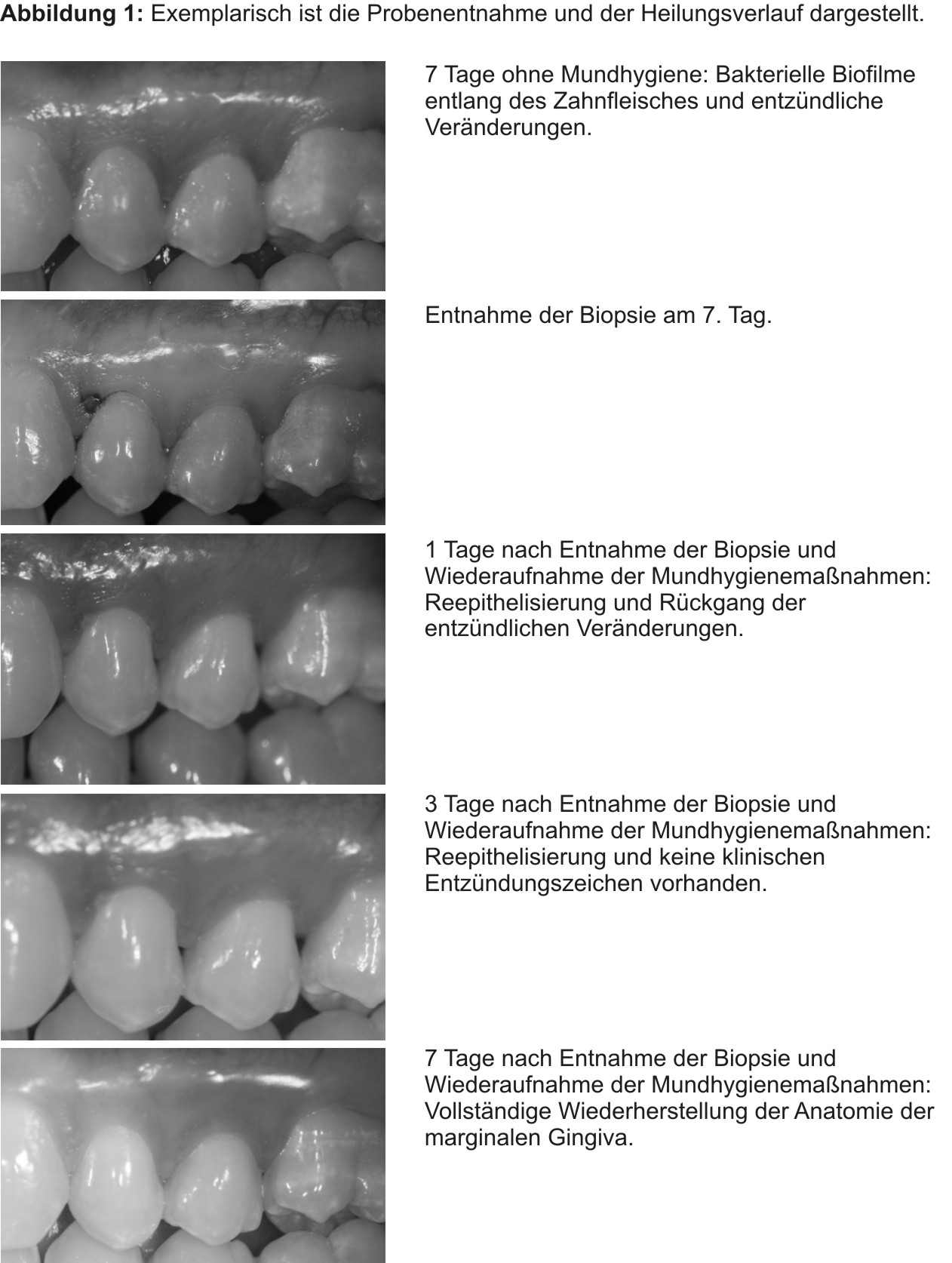


## **Darstellung der Bedeutung der zu erwartenden Ergebnisse für die Heilkunde**

Das zu erwartende Ergebnis ist eine wesentliche Voraussetzung für das Verständnis der Pathogenese der Gingivitis und Parodontitis, zwei orale entzündliche Erkrankungen mit hoher Prävalenz in der westlichen Bevölkerung. Weiter trägt die Untersuchung zum Verständnis der chemischen Barriere muko-epithelialer Grenzflächen bei gesunden und entzündlichen Bedingungen bei. Langfristig können mit diesen Erkenntnissen neue Präventions- und Therapiemöglichkeiten gefunden werden.

# **Darstellung der Untersuchungsmethoden und der Datenregistrierung**

Zu den angegebenen Zeitpunkten werden Gingivaproben entnommen und immunhistologisch gefärbt.[[2]](#footnote-3) Die Auswertung erfolgt semi-quantitativ. Während der klinischen Plaquebildung findet die Charakterisierung der entzündlichen Veränderungen durch einen Plaque- und Gingivaindex sowie über die Bestimmung der Sulkusflüssigkeit statt (Eberhard et al. 2005).

# **Darstellung der Maßnahmen, die der Sicherung der betroffenen Personen vor vermeidbaren Schäden dienen, der Einschluss- und Ausschlusskriterien sowie der Kriterien für einen Abbruch der Untersuchung**

Alle Probanden werden zu Beginn einer ausführlichen anamnestischen Befragung unterzogen. Dabei erfolgt neben der Dokumentation der Medikamenteneinnahme, dem Alkohol- und Nikotingenuss besonders auch die Erfassung der Ernährungsgewohnheiten der Probanden. Es folgt eine eingehende Inspektion der Mundhöhle, der Mundschleimhäute und der Zähne. An der Untersuchung nehmen nur gesunde Probanden ohne eine Allgemeinerkrankung teil. Die marginale Gingiva muss gesund und frei von Parodontaltaschen sein.

Vor Beginn der Behandlung und nach Abschluss der Studie erhalten alle Probanden eine professionelle Zahnreinigung, sodass sicher davon ausgegangen werden kann, dass die angesammelte Plaque vollständig beseitigt wird.

Einschlusskriterien:

- Alter 20 bis 35 Jahre
- Guter Allgemeinzustand
- Entzündungsfreie Gingiva und marginales Parodontium
- Die Patienten müssen nach entsprechender Aufklärung über Wesen, Bedeutung und Tragweite der klinischen Prüfung und der damit verbundenen Risiken, ihr jederzeit widerrufbares Einverständnis zur Teilnahme an der klinischen Prüfung erklärt haben

Ausschlusskriterien:

- Schwangere und stillende Frauen (vor Beginn der Studie muss ein Schwangerschaftstest durchgeführt werden und eine Information darüber erfolgen)
- Raucher
- Personen mit akuten oder chronischen Krankheitsbildern
- Personen, die gegenwärtig unter anderweitiger krankheitsbedingter Medikamenteneinnahme stehen, insbesondere unter nicht-steroidalen oder steroidalen Antirheumatika, Analgetika etc.
- Unversorgte kariöse Defekte, umfangreiche prothetische Versorgungen und Entzündungen des marginalen Parodontiums
- Personen mit allergischen Diathesen in der Anamnese

Abbruchkriterien:

- Jeder Proband ist – auch ohne die Angabe von Gründen – berechtigt die Studie zu jedem Zeitpunkt abzubrechen. Während der Versuchsdauer auftretende allgemeine und lokale Erkrankungen sind unverzüglich dem Versuchsleiter zu melden und führen zum Ausschluss aus der Studie.

- Vorzeitiger Ausschluss wird dokumentiert und der Proband ersetzt.

# **Angaben über den Abschluss einer Patienten- bzw. Probandenversicherung**

Eine Probandenversicherung wird abgeschlossen.

# **Angaben darüber, ob ( nur ) volljährige oder ( auch ) minderjährige ( unter 18 Jahren ) Patienten oder Probanden oder Frauen im gebärfähigen Alter untersucht werden sollen**

Es werden nur volljährige Patienten untersucht. Frauen im gebärfähigen Alter dürfen nur an der Studie teilnehmen, wenn Sie vor Beginn der Studie einen Schwangerschaftstest durchgeführt haben.

# **Erklärung ob und ggf. wo bereits parallele Anträge bei anderen Ethik-Kommissionen eingereicht wurden**

Es wurden keine anderen Anträge zu diesem Forschungsvorhaben eingereicht.

Bissell, J., Joly, S., Johnson, G. K., Organ, C. C., Dawson, D., McCray, P. B., Jr. & Guthmiller, J. M. (2004). Expression of beta-defensins in gingival health and in periodontal disease. *J Oral Pathol Med*, **33**, 278-285.

Chung, W. O., Dommisch, H., Yin, L. & Dale, B. A. (2007). Expression of defensins in gingiva and their role in periodontal health and disease. *Curr Pharm Des*, **13**, 3073-3083.

Diamond, D. L., Kimball, J. R., Krisanaprakornkit, S., Ganz, T. & Dale, B. A. (2001). Detection of beta-defensins secreted by human oral epithelial cells. *J Immunol Methods*, **256**, 65-76.

Dommisch, H., Acil, Y., Dunsche, A., Winter, J. & Jepsen, S. (2005). Differential gene expression of human beta-defensins (hBD-1, -2, -3) in inflammatory gingival diseases. *Oral Microbiol Immunol*, **20**, 186-190.

Dunsche, A., Acil, Y., Siebert, R., Harder, J., Schroder, J. M. & Jepsen, S. (2001). Expression profile of human defensins and antimicrobial proteins in oral tissues. *J Oral Pathol Med*, **30**, 154-158.

Eberhard, J., Menzel, N., Dommisch, H., Winter, J., Jepsen, S. & Mutters, R. (2008). The stage of native biofilm formation determines the gene expression of human beta-defensin-2, psoriasin, ribonuclease 7 and inflammatory mediators: a novel approach for stimulation of keratinocytes with in situ formed biofilms. *Oral Microbiol Immunol*, **23**, 21-28.

Eberhard, J., Reimers, N., Dommisch, H., Hacker, J., Freitag, S., Acil, Y., Albers, H. K. & Jepsen, S. (2005). The effect of the topical administration of bioactive glass on inflammatory markers of human experimental gingivitis. *Biomaterials*, **26**, 1545-1551.

Loe, H., Theilade, E. & Jensen, S. B. (1965). Experimental Gingivitis in Man. *J Periodontol*, **36**, 177-187.

Ramberg, P., Sekino, S., Uzel, N. G., Socransky, S. & Lindhe, J. (2003). Bacterial colonization during de novo plaque formation. *J Clin Periodontol*, **30**, 990-995.

1. Siehe Amendment Kardiologie und Biobank [↑](#footnote-ref-2)
2. Siehe Amendment Kardiologie und Biobank [↑](#footnote-ref-3)
